# Antibody Protection Reveals Extended Epitopes on the Human TSH Receptor

**DOI:** 10.1371/journal.pone.0044669

**Published:** 2012-09-05

**Authors:** Rauf Latif, Avelino Teixeira, Krzysztof Michalek, M. Rejwan Ali, Max Schlesinger, Ramkumarie Baliram, Syed A. Morshed, Terry F. Davies

**Affiliations:** 1 Department of Medicine, Mount Sinai School of Medicine, New York, New York, United States of America; 2 Thyroid Research Unit, James J. Peters VA Medical Center, New York, New York, United States of America; 3 Proteomics Laboratory, Department of Medicine, Mount Sinai School of Medicine, New York, New York, United States of America; 4 Department of Endocrinology, Metabolism and Internal Diseases, Poznan University of Medical Sciences, Poznan, Poland; Cardiff University, United Kingdom

## Abstract

Stimulating, and some blocking, antibodies to the TSH receptor (TSHR) have conformation-dependent epitopes reported to involve primarily the leucine rich repeat region of the ectodomain (LRD). However, successful crystallization of TSHR residues 22–260 has omitted important extracellular non-LRD residues including the hinge region which connects the TSHR ectodomain to the transmembrane domain and which is involved in ligand induced signal transduction. The aim of the present study, therefore, was to determine if TSHR antibodies (TSHR-Abs) have non-LRD binding sites outside the LRD. To obtain this information we employed the method of epitope protection in which we first protected TSHR residues 1–412 with intact TSHR antibodies and then enzymatically digested the unprotected residues. Those peptides remaining were subsequently delineated by mass spectrometry. Fourteen out of 23 of the reported stimulating monoclonal TSHR-Ab crystal contact residues were protected by this technique which may reflect the higher binding energies of certain residues detected in this approach. Comparing the protected epitopes of two stimulating TSHR-Abs we found both similarities and differences but both antibodies also contacted the hinge region and the amino terminus of the TSHR following the signal peptide and encompassing cysteine box 1 which has previously been shown to be important for TSH binding and activation. A monoclonal blocking TSHR antibody revealed a similar pattern of binding regions but the residues that it contacted on the LRD were again distinct. These data demonstrated that conformationally dependent TSHR-Abs had epitopes not confined to the LRDs but also incorporated epitopes not revealed in the available crystal structure. Furthermore, the data also indicated that in addition to overlapping contact regions within the LRD, there are unique epitope patterns for each of the antibodies which may contribute to their functional heterogeneity.

## Introduction

Graves’ disease is a classic example of a disease where autoantibody mediated receptor activation is the major cause of the clinical phenotype. The target of these autoantibodies is the thyroid stimulating hormone receptor (TSHR), a G protein-coupled receptor present on the plasma membrane of thyrocytes (and other extra-thyroidal cells including fibroblasts, adipocytes and bone cells) [1], [2] which is required to carry out many of the specialized functions of the thyroid gland [3]. The TSHR belongs to the subfamily of glycoprotein receptors that display a bipartite structure consisting of a large amino terminal extracellular domain (ECD) responsible for high affinity hormone binding and a serpentine membrane terminal portion which is a characteristic of the opsin family of G proteins [4]. The ECD consists of a well characterized leucine rich domain (LRD) starting from residues 22–260, after removal of the signal peptide, and encompassing 10 leucine rich repeats, followed by a region of approximately 130 amino acids that has been termed the “hinge region” [2], [5], [6]. This latter region has, to date, defied crystallization, and it has not been possible to model since it lacks homology to any known structure. The TSHR not only has the longest hinge region of similar receptor structures but it also harbors a unique 50 amino acid peptide which is deleted by proteolysis (cleavage) leading to a final bipartite receptor structure [7], [8]. These post-translational changes result in an extracellular ligand sensing α- (or A) subunit and a membrane embedded β- (or B) subunit which are joined by covalent bonds [8], [9].

It was first believed that the LRD region of the ectodomain was the main and only interacting site for TSH and TSHR autoantibodies but several studies have now shown that non- LRD binding sites are also involved in receptor activation [10], [11], [12] There is now an emerging concept to explain signaling at the TSH receptor as a consequence of its post-translational structural alterations which also includes multimer formation [13], [14]. Recent studies have shown that the hinge region is not an inert scaffold but harbors positive and negatively charged residues which actively interact with the α and β subunit residues of the TSH ligand itself [11] and stabilizes the receptor conformation that is required for signal transduction. Indeed the higher potency of porcine and bovine TSH preparations compared to recombinant human TSH has been explained by their interaction with non-LRD regions [15], and this has been confirmed by studies with mutated hinge regions [16].

**Figure 1:**
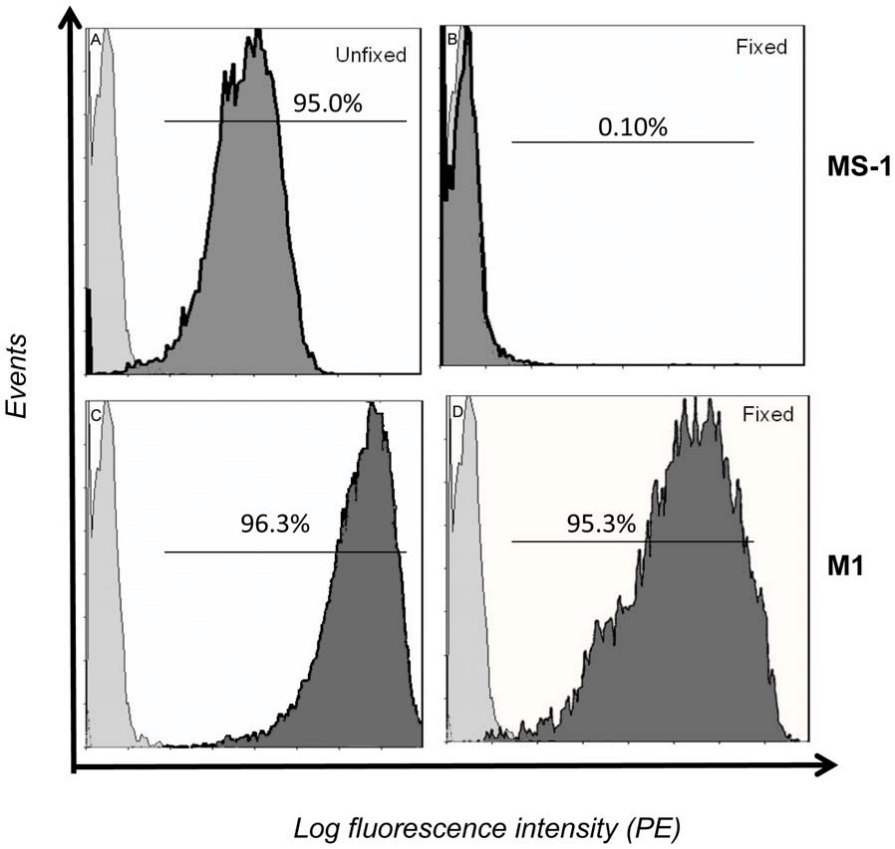
Conformational identity of TSHR antibodies. To show conformational dependence of stimulating TSHR antibodies, CHOTSHR expressing the TSHR were treated with 4% paraformaledhyde for 30 minutes at room temperature and after washing twice with PBS the cells were incubated with 1 ug of antibody for 1×10^6^ cells/tube of TSHR-mAbs MS-1 or M1 in FACS staining buffer containing 2% FBS (Panels A and B). In parallel, non-paraformaldehyde treated (unfixed) cells were also incubated with the same concentration of MS-1 and M1 antibodies in FACS staining buffer containing 2% FBS (Panels C and D). Treatment of cells expressing the human TSHR with paraformaldehyde destroyed the ability of conformational antibody MS-1 to be recognized whereas the antibody that recognized a linear epitope (M1) was not altered by the fixation.

Crystallization of FSH bound to the FSH receptor [17] revealed the precise sites of binding by a glycoprotein hormone to the concave surface of the LRD and this allowed the first comparative modeling of TSH-TSHR interaction [18], [19]. Using this information and the subsequent TSHR-M22-Fab crystal structure, it was possible to identify receptor residues that are important for stimulating TSHR antibody binding [20], [21]. These studies extended our previous understanding of the TSH binding pocket as delineated using a series of monoclonal antibodies [22]. The recent crystallization of a TSHR blocking antibody has added further insight into these interactions by suggesting a different orientation of the LRD that is captured by blockers over that of the stimulators [23]. How these auto antibodies activate or block the receptor by their presumed structural influence is still not fully understood.

Knowing the complete tripartite structure of the TSHR (LRD, hinge and TMD) and their relative orientation to one another would help us to better understand ligand and autoantibody binding and how this might lead to receptor activation. However, the lack of a crystal structure for the entire TSHR complex has led us to resort to conformational epitope mapping by other means. Discontinuous epitopes are dependent on structural conformation of the protein may complicate the interpretation of point directed mutagenesis and overlapping peptide analysis. In the case of mutagenesis, studies that demonstrate loss of binding may not necessarily equate with the identity of the epitope but may result in unrelated structural changes. Another approach, that of steric protection of amino acids at the interface of a monoclonal antibody followed by limited proteolysis and matrix assisted laser desorption/ionization mass spectrometry (MALDI-MS) has been successfully used to study a few conformational epitopes [24]. We have, therefore, used this latter approach to map the binding residues of TSHR antibodies (blocking and stimulating) which bind to the TSHR ectodomain and have identified their non-LRD binding sites.

**Figure 2:**
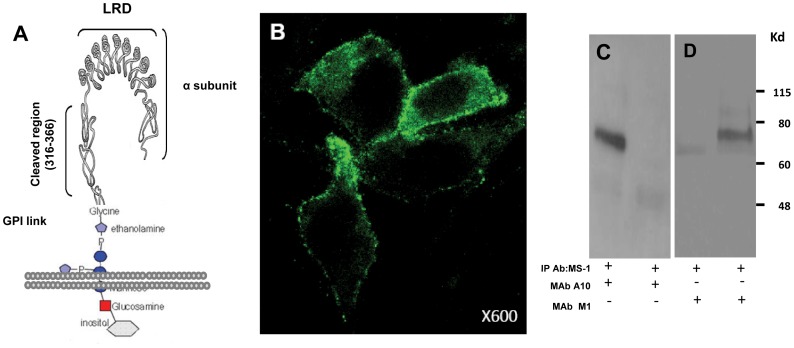
TSHR ectodomain linked to GPI as a source of antigen. **Panel A -** The source of the native antigen for epitope protection studies was the entire TSHR ectodomain (ECD) protein which was anchored to the plasma membrane via a GPI linker. This link could be cleaved by phospholipase C treatment to obtain large amounts of purified receptor ectodomain. **Panel B -** Surface expression of the TSHR-GPI construct on CHO cells is illustrated by staining with TSHR-mAb M1 directly conjugated to Alexa 488. **Panel C** - Immunoprecipitation of solubilized receptors from total membrane preparations obtained from GPI-TSHR-ECD cells precipitated using MS-1 (2 ug/ml) (lane 1) and probed with an antibody to the N-terminus of the receptor (A10). A specific band corresponding to 74KD protein was observed in lane 1 whereas no immunoprecipitation was observed with MS-1 using membranes prepared from untransfected CHO cells (lane 2) and probed with an antibody directed to amino terminus of receptor (A10, residues 22–41). **Panel D -** PI-PLC digest obtained from GPI-TSHR-ECD cells immunoprecipitated with MS-1 (2 ug/ml) (lane 2) and from GPI-TSHR-ECD cells untreated with PI-PLC (lane 1). A specific band corresponding to a 74KD protein was observed in lane 2 compared to a faint lower weight band obtained with vehicle alone (lane 1). The faint band maybe is the result of CHO-TSHR cell breakdown that resulted in some binding of TSHR-mAb.

## Materials and Methods

### Antibodies Employed

MS-1 is our well characterized hamster derived monoclonal stimulating TSHR antibody [25], M-22 is a human stimulating monoclonal TSHR antibody ) [20], [26] (kind gift from RSR Ltd, Cardiff, UK) and Tab-8, is a hamster derived TSH blocking monoclonal TSHR antibody [27]. All three of these monoclonal antibodies had conformational epitopes on the TSHR as evidenced by flow cytometric analysis. In contrast, murine monoclonal TSHR-Abs A10 (Serotec,CA), M1 (RSR 1) and M4 (RSR 4) with linear epitopes at residues 22–41, 381–385 and 322–342 respectively, were used as control TSHR antibodies (also gifts from RSR Ltd, Cardiff, UK). An additional control was normal hamster IgG (Jackson Immuno Research Inc, CA).

### Preparation of TSHR Ectodomain

Stable lines of GPI-TSHR transfected cells used in this study were generated by transfecting a GPI linked TSHR ectodomain (kindly provided by Dr. A.P Johnstone, St. George's Hospital Medical School, UK) into CHO cells [28]. These cells were maintained in Ham F12 with 10% FBS and 100 IU of penicillin/streptomycin as described earlier. ECD was prepared directly from these cells. Briefly, four 150 mm dishes of CHO-TSHR cells were cultured in Ham’s F12 medium with 10% fetal bovine serum and 100 µU/ml of penicillin and streptomycin until the cells were 90% confluent. 10^8^ GPI -TSHRT-ECD cells were washed three times with 1X PBS (without Mg/Ca ++) and incubated with PI-PLC (0.1 unit) in 500 ul of PI-PLC buffer for 10 min at 4C and the supernatant harvested after pelleting the cells by centrifugation.

### Flow Cytometry and Binding

CHO-TSHR cells (28) and GPI-TSHR-ECD cells (as described above) before and after PI-PLC treatment, the cells were washed twice with PBS pH 7.4 at room temperature and distributed as 1×10^6^ cells per tube for staining. 1 ug/ml of TSHR specific antibody was then added to these treated cells and incubated at room temperature for 1 hr. After washing twice with FACS buffer, the cells were stained with detecting antibody (anti mouse Fab’ conjugated to FITC) and the resulting loss of ectodomain binding was observed.

**Figure 3:**
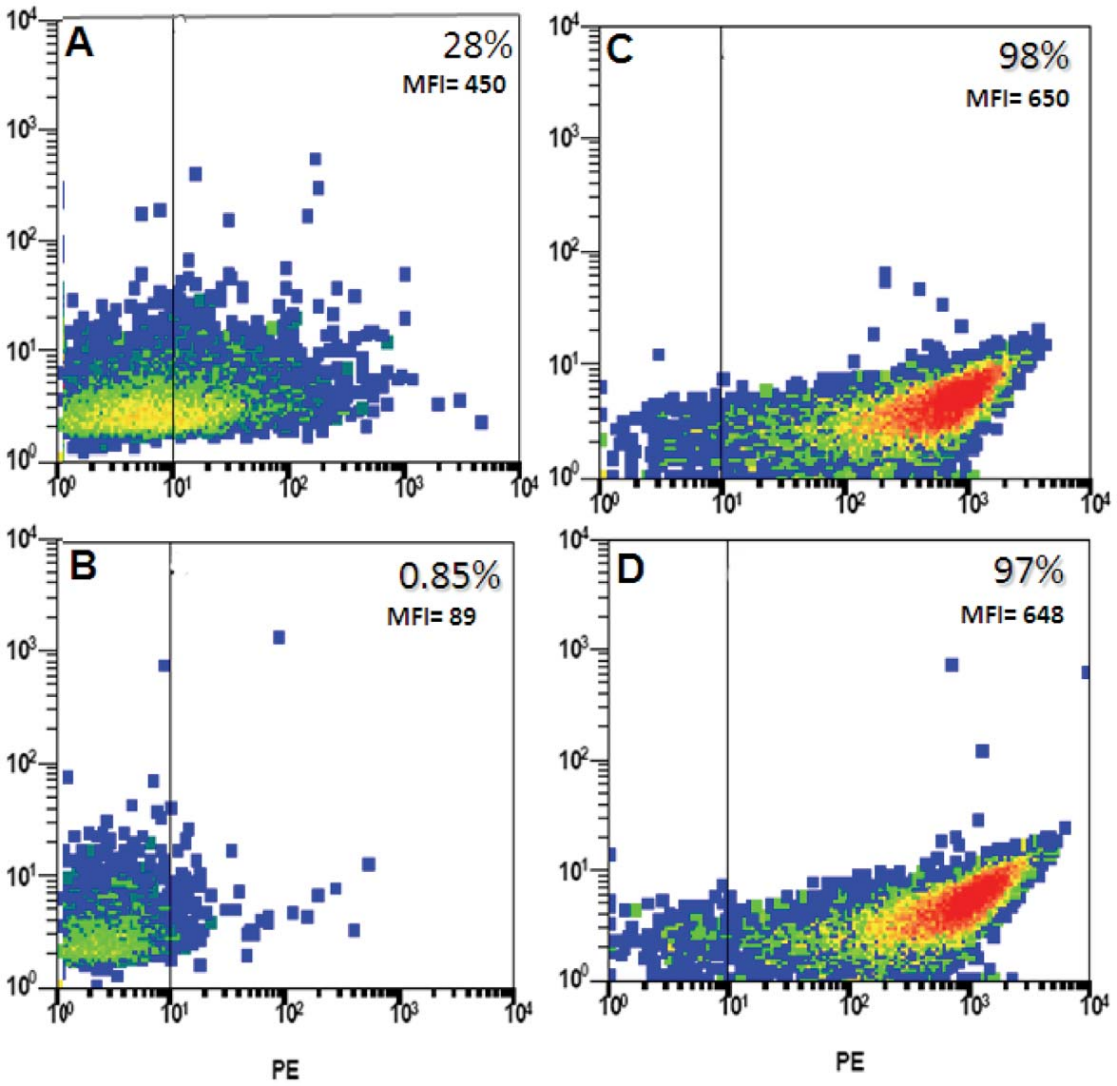
Purification of the TSHR ectodomain. The cells expressing the GPI-TSHR-ECD receptors (panels A and B) or wild type CHO-TSHR (panels C and D) were digested for 10 minutes at 4C with 0.1 units of PLC in sterile PBS pH 7.4 and the supernatant was collected by centrifugation. The cells were checked before and after digestion by FACS for release of the ECD. An 80% decrease in MFI was observed after PLC treatment of the GPI-TSHR-ECD cells (A and B) whereas control CHO-TSHR cells expressing a non-GPI linked TSHR subjected to the same conditions did not show a decrease (C and D).

### Co-immunoprecipitation with MS-1

Ectodomain preparations from GPI-TSHR-ECD cells, and solubilized cell membranes prepared from CHO-TSHR cells as control (13), were immunoprecipitated with MS-1. Briefly, the PI-PLC eluted fraction obtained from GPI-TSHR-ECD preparations was precipitated by the addition of 2 µg/ml of hamster monoclonal antibody MS-1 for 3 hr at 4°C. This was followed by a pull down of the immune complex with protein G sepharose beads. The beads were reduced by treating them with 5x sample buffer containing 100 mM of DTT for 45 minutes at 50°C. The immunoprecipitates were resolved on 12% SDS-PAGE and electro-blotted onto PVDF membranes. Membranes were blocked with 5% dried skimmed milk in TBS with 0.05%Tween20 (TBST) and then probed with two different antibodies recognizing two regions of the TSHR ectodomain (1) An antibody that recognized the extreme N terminus of the protein (A10 - recognizing residues 22–41) and (2) The M1 TSHR antibody (to residues 381–385) as the second probe. Washed membranes were then incubated with 1∶10,000 of secondary antibody (anti-mouse HRP) for 1 hr at room temperature. After final washing, bound secondary antibodies were visualized using enhanced chemiluminescence (Super Signal ECL, Pierce, and IL).

**Figure 4:**
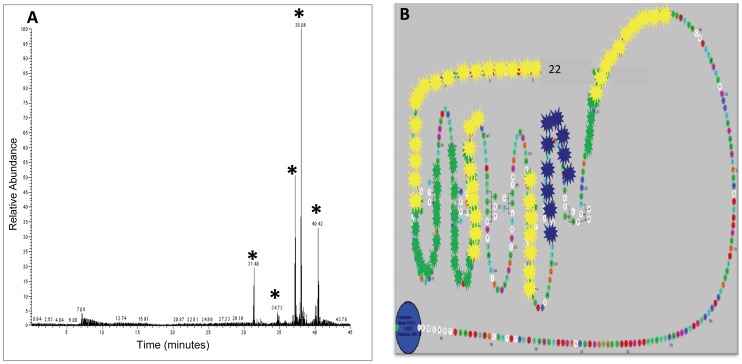
Mass spectrometry and mapping of protected epitopes of MS-1 and M22. **Panel A -** The antibody protected peptides were eluted and fed into the mass spectrometer which scanned the incoming peptides based on user defined criteria in the database and was able to select TSHR specific tryptic peptides. By this method we obtained five major peaks for peptides protected by MS-1(marked by an asterisk). The captured peptides were dissociated in an ion spray and the masses of the component amino acids measured and identified. Additional profiles are provided as Supplementary Material. **Panel B -** The common regions, indicated by the yellow stars, and regions that were unique to each of the stimulating antibodies, indicated by the blue (M22) and green (MS-1) stars, illustrate the relative similarity and difference between the protected regions of these two antibodies. The exact contact residues for M22 (red circles) obtained from the crystal structure [20] when overlaid on this model identified ∼60% of the reported residues. The regions on the leucine rich repeats contacted by MS-1 corresponded to LRD 1, 4, 7, 8 and 9. In addition two regions outside the LRD were protected, one region was on the amino terminus starting and the second large region in the N- terminus of the hinge region. Similarly the regions contacted by M22 corresponded to LRD 1, 2, 3, 4 and 7. As seen with MS-1, the amino terminus and hinge region epitopes were also protected. These epitope protection studies were repeated three times (n = 3) for each antibody.

**Figure 5:**
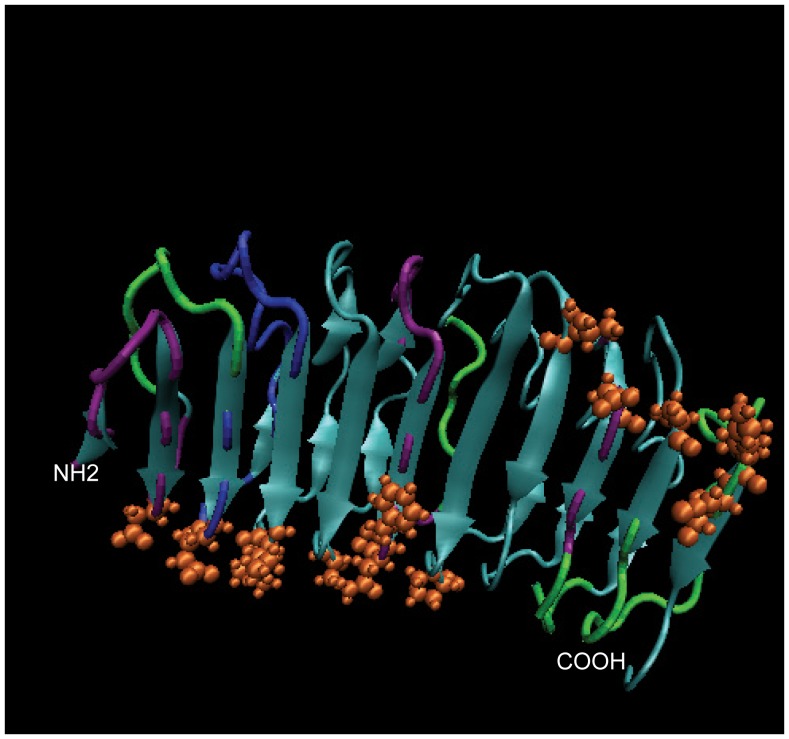
Mapping similar and unique antibody protected regions on TSHR LRD 3D structure. The 3D structure of the TSHR ectodomain, based on Sanders et al (20), illustrating the apparently protected regions following binding of MS-1 and M22 previously shown on the snake plot (Figure 4B). Purple represents common protected regions, Green the MS-1 unique regions, and blue the M22 unique regions. The major TSH binding residues are also shown in orange.

**Figure 6:**
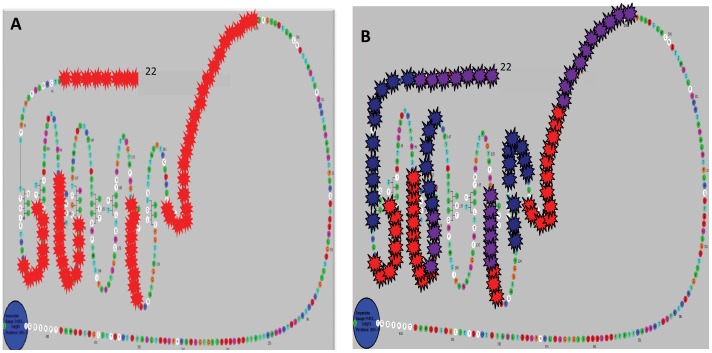
The protected epitopes of TAb-8 and comparison of it to stimulating antibody epitopes. **Panel A:** Though the epitopes of TAb-8 blocking TSHR-Ab looked similar to the stimulating antibodies there were, once again, some major differences in the residues contacted. Unlike the stimulators, the epitopes for this antibody in the LRD region were restricted to LRD 2, 3, 4 and 7 (red stars). The protected amino terminus segment was shorter and the hinge region segment was longer and extended to residue 291. **Panel B:** The comparison of blocking antibody (TAb-8) to that of stimulating antibodies MS-1 and M22 showed some unique regions (red stars) which may account for its ability to change the conformation of the receptor rendering it inert to TSH binding. The binding residues of stimulating TSHR antibody MS-1 are shown with blue stars and with purple stars. The purple stars indicate where both the blocker and the stimulator both protected residues.

### Antibody Protection Assay and Mass Spectrometry

Having established that the conformation of antigen was retained in the purified TSHR ECD preparations by the co-immunoprecipitation experiments, we carried out antibody protection of the TSHR as follows:

1. We incubated 1 ug with a variety of TSHR monoclonal antibodies (as described above) with 0.1 ug of purified ECD in 10 mM H_4_HCO_3_ buffer pH8.0 at 37°C for 1 h.
2. This antibody-antigen complex was then incubated with Protein G conjugated sephraose beads and incubated further for another 2 h in mM NH_4_HCO_3_ buffer at 37°C. At the end of this incubation, the beads were washed 2 times with buffer.
3. The antigen bound to the cross-linked antibody was then subjected to limited proteolysis on mini columns with immobilized trypsin (10 ng/ml) (Thermo Scientific, IL) for 3 h at 37°C which was empirically determined.
4. On completion of digestion, the beads were removed by centrifugation and washed stringently several times with H_4_HCO buffer pH 8.0.
5. The fragments of the antigen bound to receptor were eluted using 10% acetonitrile/0.1% formic acid and eluates were lyophilized and dissolved in 10% acetonitrile/0.1% FA organic solvent and analyzed by LC/MS (LTQ Thermo) with the mass spectrometer set to collect and analyze all peptides with masses equivalent to all ECD tryptic peptides.

### Database Searching and Analysis of the MS Spectra

Mass spectometry data were searched against a non-redundant database and a human TSHR protein database using the SEQUEST algorithm from the BioWorks suite of mass analysis software (Thermo Scientific, IL).

## Results

### Conformational Identity of Monoclonal Antibodies

The conformational nature of the TSHR antibody epitopes was initially confirmed prior to performing epitope protection studies. Cells expressing the TSHR (CHO-TSHR) were fixed briefly with 4% paraformaldehyde as described in the legend to Figure 1. The TSHR-Ab-MS-1 lost its ability to bind to the receptor as indicated by the decreased fluorescence intensity on flow cytometry when compared with unfixed cells where the antibody retained its binding capacity (**Figures 1A and 1B**). The binding of a TSHR-mAb (M1), that recognized a linear epitope, remained unaltered by fixation of CHO-TSHR cells (**Figures 1C and 1D**). Similar data were obtained for human stimulating antibody M22 and hamster blocking antibody TAb-8 (data not shown).

### Cell Surface Expression of TSHR ECD-GPI

The entire ectodomain of the TSH receptor (1–412aa) was linked to GPI (**Figure 2A** **)** and the construct expressed on CHO cells. The surface expression of the TSHR ectodomain was then ascertained histologically by immunofluorscent staining using TSHR-Ab-M1(RSR1 or M1) conjugated to Alexa 488 (**Figure 2B**).

### Bulk Receptor Preparation

Purified ECD was prepared by digesting CHO-TSHR-ECD with PI-PLC. FACS analysis (**Figure 3**) before (Panels A and C) and after digestion (Panels B and D) indicated a marked reduction in the percentage positive cells after digestion (28% versus 0.85%) and marked reduction in the mean fluorescence intensity (MFI) (450 to 89 arbitrary fluorescence units) with the TSHR-ECD-GPI cells. The control CHO-TSHR (WT) cells lacking the GPI linked receptor retained the same positivity before and after PI-PLC digestion (98% and 97%) with no significant change in their MFI.

### Antigenicity of Purified TSHR Ectodomain

TSH receptors released from the cell surface might have lost binding to antibody because of conformational changes in the released protein and/or degradation of the protein. Therefore, retention of antibody binding to the TSHR ectodomain released from the surface of the transfected CHO cells was critical to the success of the planned epitope protection studies. To be sure that the TSHR-ECD released from the surface of cells retained the ability to bind to conformationally dependent TSHR-Abs we performed co-immunoprecipitation in total membranes (**Figure 2C** **)** and TSHR ECD released from the cell surface (**Figure 2D**). As shown by the immunoblotting, the conformationally dependent TSHR-mAb MS-1 was still capable of binding to TSHR ectodomain preparations even after PI-PLC treatment.

### Identification of Conformational Epitopes of Stimulating TSHR Antibodies

Mass spectrometric analysis of peptide fragments obtained from the epitope protection of stimulating TSHR antibody MS-1 yielded five major epitopic regions throughout the TSHR ectodomain (**Figure 4A** **)**. Control antibodies revealed no identifiable peptide fragments. The results were best illustrated by overlaying the regions obtained onto a snake plot of the entire TSHR-ECD (**Figure 4B**). The five LRD regions that were recognized corresponded to LRRs 1, 4, 7, 8 and 9. There were also two regions recognized by MS-1 outside the LRD - a segment of ectodomain between position methionine #22 (the first amino acid after the signal peptide) and S53 encompassing the N-terminal cysteine cluster 1 and a second region of 21 amino acids from R274 to Q294 in the amino terminus of the hinge region. Analysis of the human stimulating TSHR-mAb M22 protecting the TSHR gave a similar mass spectrometry profile with residues encompassing the major LRD region, the amino terminus of the receptor ectodomain, and a large segment of the hinge region (**see Figure S1**). The snake plot (**Figure 4B**) revealed five major regions in the LRD portion covering LRRs 1, 2, 3, 4 and 7. Interestingly, in the case of M22, the segments that were protected in the LRD included five β pleats and four of these were restricted to the amino half of the LRD. Like MS1, M22 also covered the N terminal cysteine cluster and a larger region of the hinge region starting at L263 and ending at K291. Overlaying residues (marked by red circles) obtained by crystallization of M22 Fab, onto the snake plot, showed that there was a ∼60% identification of the reported residues by using the epitope protection method (**Figure 4B**). On comparing our hamster stimulating antibody MS-1 and the human stimulating antibody M22 we found several unique and common epitopes between the two stimulating TSHR antibodies in the LRD region when mapped on the 3D structure (**Figure 5**). This form of analysis showed that the amino and hinge regions of the TSHR were common epitopes to both stimulating antibodies with almost a complete overlap for these regions. The segments that were unique to these two antibodies were mostly restricted to the LRD region.

### Identification of the Conformational Epitopes of a Blocking TSHR-Ab

To identify if blocking TSHR-Abs had any major differences in epitope recognition we analyzed the protected epitopes of TSHR-mAb TAb-8. This is a hamster monoclonal antibody to the TSHR with powerful TSH blocking activity [27]. Mapping the identified residues by mass spectrometry of this antibody on the snake plot showed that it had only 3 major segments of contact on the LRD portion of the receptor, with LRRs 2, 3, 4 and 7 contacted (**Figure 6A**). However, the non-LRD protected segments had again two regions - the amino terminus segment ranged from G21 to V39 encompassing the cysteine loop 2 and the hinge region from L252 all the way to R292, which like the stimulating antibody had the SHCCAF motif enclosed within this region.

### Comparing Stimulating and Blocking TSHR-Abs

The blocking antibody epitopes, when compared to the stimulating epitopes, showed binding to a shorter amino terminus segment of the receptor and a larger region of the hinge region encompassing the known critical residue 255 [20], [29] which has been shown to be needed for normal receptor signaling. On comparing the blocker to the stimulating antibodies we saw some common regions but also several unique regions to the TSHR blocker (**Figure 6B**).

## Discussion

The precise mechanism by which the immune system is activated in its response to the TSHR antigen in Graves’ disease remains unclear but it is now known that the various pathogenic auto antibodies to the TSHR activate or deactivate the receptor by binding to contiguous, non-contiguous or to both types of epitopes on the extracellular region of the receptor [30], [31], [32]. Conventionally, conformational antibody epitopes have been studied using molecular modeling and site directed mutagenesis [32].However, using the mutagenesis approach discontinuous epitope of two TSH receptor stimulating mouse monoclonal antibody has been precisely mapped to the amino-terminal portion of the LRD [33] Although these methods can be robust approaches their reliability and applicability to conformational epitopes is questionable since mutagenesis may interfere with the three dimensional tertiary structure of the protein. However studies by Cornelis et al [34] have shown that ectodomain of TSHR receptor prepared by PI-PLC has affinity not only to TSH but also auto antibodies suggesting this preparation is close native state. Our results described here using the antibody protection technique, coupled with mass spectrometry, indicated that the conformational epitopes on the TSHR ectodomain which are recognized by stimulating and blocking antibodies are not confined to the LRD region of the receptor.

We currently have most insight into the contact residues of human stimulating antibody (M22) and human blocking antibody (K1–70) following crystallization of the TSHR ectodomain bound to the Fab fragments of these antibodies [23]. However, the crystal structures employed residues 22–260 and thus revealed the binding characteristics of the antibodies only within the LRD region. We knew previously from several earlier studies that the LRD region is the critical binding site for TSH [35] and is known to cradle the TSH binding pocket [22]. TSH binds primarily to the concave surface of the LRD region, making contact with almost all the 10 β pleated sheets of this domain in a “hand clasped” fashion [20], [23] similar to other glycoprotein hormones [17], [36]. Similarly studies using stimulating and blocking monoclonal antibodies and patient IgGs have also pointed to the LRD as a major region for initiation of stimulation or inhibition of ligand activation [2], [20], [21], [23], [27]. Thus, undoubtedly the LRD is the major part of the receptor for binding and perhaps also signal transduction but it is certainly not the only region. The hinge region of the TSHR encompasses residues 277–412 and is, therefore, outside of the LRD but has recently been shown to be important for ligand binding and signaling [10], [11], [12], [16]. This region, which was thought earlier to be an inert scaffold joining the LRD to the transmembrane region, is now known to contain residues that are critical for ligand binding and signaling. It has also been shown that a tyrosine residue located downstream of the C terminus cystein cluster in this region is mandatory for high affinity TSH binding and activation of the receptor [37] Studies from our laboratory have shown that neutral antibodies directed to the cleaved region (316–366) are capable of activating the receptor leading to non-cAMP dependent signaling [30]. In agreement with these studies, mutation analysis of the hinge region of the receptor demonstrated an extended hormone binding site that is also involved in receptor activation [29], [38]. Mutation studies within the LRD itself have shown that such binding sites can vary, suggesting subtle differences in the amino acids that make contact with the different stimulators and blockers. Thus, agonistic or antagonist activity may depend more on the nature of the interacting residues than on the extent of the interacting surface although the exact triggers for these actions have yet to be found.

Since the autoantibody epitopes are conformation-dependent, we examined the epitopes of TSHR antibodies after minimal perturbation of the native structure which also allowed us to delineate antibody binding regions outside the LRD. Using this approach we examined two stimulating antibodies (MS-1 and M22) and one blocking monoclonal antibody (Tab-8) and compared their binding profiles. When we examined the epitopes of the high affinity human stimulating antibody M22 using the antibody protection technique, the peptide profile revealed five major regions that the antibody contacted within the ectodomain. Mapping the amino acid sequences of these different peaks on a snake plot of the entire ECD (1–412 aa) revealed major regions on LRD 1, 2, 3, & 4. The analysis identified two regions outside the LRD. The first was at the amino terminus of the receptor starting immediately after the signal peptide and the second site was the N terminus of the hinge which also encompassed a previously described activation motif (SHCCAF). In the LRD region we could define 14 of the 23 (61%) contact residues described in the crystal structure of M22 in a complex with the TSHR ECD [23]. Similarly, analysis of stimulating monoclonal antibody MS-1 [25] also indicated five major regions on the LRD; regions 1, 4, 7, 8 & 9. These differences in the contact residues (<50%) may also account for the affinity difference between these monoclonal antibodies. The 2 none LRD regions contacted by this antibody were similar to M22 except for minor differences in the number of residues contacted in the hinge region. Similar data were obtained for blocking mAb Tab-8 although the degree of binding to the LRD region was much less. More antibodies need to be examined before we can draw any broad conclusions concerning these differences in the protected LRDs. However, the current technique using antibody protection from trypsin digestion alone has led us to finding large regions encompassing the antibody epitope that may have arisen due the tertiary and quaternary structure of the protein. A finer mapping of these regions to determine the minimum epitopes will require further digestion of the exposed receptor residues using amino and carboxyl peptidases [39], [40], [41].

## Supporting Information

Figure S1 **The peptide sequence of the entire TSH receptor ECD is shown in black letters in the top and bottom panels.** The MS-1 protected peptides shown in the top panel (marked in red) and M22 protected peptide sequences shown in bottom panel (marked in green).
(TIF)

## References

[1] Davies T, Marians R, Latif R (2002) The TSH receptor reveals itself. J Clin Invest 110: 161–164. 12122107 10.1172/JCI16234 PMC151075

[2] Rapoport B, Chazenbalk GD, Jaume JC, McLachlan SM (1998) The thyrotropin (TSH) receptor: interaction with TSH and autoantibodies. Endocr Rev 19: 673–716. 9861544 10.1210/edrv.19.6.0352

[3] Vassart G, Dumont JE (1992) The thyrotropin receptor and the regulation of thyrocyte function and growth. Endocr Rev 13: 596–611. 1425489 10.1210/edrv-13-3-596

[4] Salon JA, Lodowski DT, Palczewski K (2011) The significance of G protein-coupled receptor crystallography for drug discovery. Pharmacol Rev 63: 901–937. 21969326 10.1124/pr.110.003350 PMC3186081

[5] Mizutori Y, Chen CR, Latrofa F, McLachlan SM, Rapoport B (2009) Evidence that shed thyrotropin receptor A subunits drive affinity maturation of autoantibodies causing Graves’ disease. J Clin Endocrinol Metab 94: 927–935. 19066298 10.1210/jc.2008-2134 PMC2681282

[6] Russo D, Chazenbalk GD, Nagayama Y, Wadsworth HL, Seto P, et al (1991) A new structural model for the thyrotropin (TSH) receptor, as determined by covalent cross-linking of TSH to the recombinant receptor in intact cells: evidence for a single polypeptide chain. Mol Endocrinol 5: 1607–1612. 1779967 10.1210/mend-5-11-1607

[7] Chazenbalk GD, Tanaka K, Nagayama Y, Kakinuma A, Jaume JC, et al (1997) Evidence that the thyrotropin receptor ectodomain contains not one, but two, cleavage sites. Endocrinology 138: 2893–2899. 9202233 10.1210/endo.138.7.5259

[8] Loosfelt H, Pichon C, Jolivet A, Misrahi M, Caillou B, et al (1992) Two-subunit structure of the human thyrotropin receptor. Proc Natl Acad Sci U S A 89: 3765–3769. 1570295 10.1073/pnas.89.9.3765 PMC525571

[9] Couet J, Sar S, Jolivet A, Hai MT, Milgrom E, et al (1996) Shedding of human thyrotropin receptor ectodomain. Involvement of a matrix metalloprotease. J Biol Chem 271: 4545–4552. 8626810 10.1074/jbc.271.8.4545

[10] Kleinau G, Jaschke H, Neumann S, Lattig J, Paschke R, et al (2004) Identification of a novel epitope in the thyroid-stimulating hormone receptor ectodomain acting as intramolecular signaling interface. J Biol Chem 279: 51590–51600. 15345720 10.1074/jbc.M404748200

[11] Mizutori Y, Chen CR, McLachlan SM, Rapoport B (2008) The thyrotropin receptor hinge region is not simply a scaffold for the leucine-rich domain but contributes to ligand binding and signal transduction. Mol Endocrinol 22: 1171–1182. 18218728 10.1210/me.2007-0407 PMC2366178

[12] Mueller S, Kleinau G, Jaeschke H, Paschke R, Krause G (2008) Extended hormone binding site of the human thyroid stimulating hormone receptor: distinctive acidic residues in the hinge region are involved in bovine thyroid stimulating hormone binding and receptor activation. J Biol Chem 283: 18048–18055. 18441013 10.1074/jbc.M800449200

[13] Latif R, Graves P, Davies TF (2001) Oligomerization of the human thyrotropin receptor: fluorescent protein-tagged hTSHR reveals post-translational complexes. J Biol Chem 276: 45217–45224. 11535591 10.1074/jbc.M103727200

[14] Latif R, Michalek K, Davies TF (2010) Subunit interactions influence TSHR multimerization. Mol Endocrinol 24: 2009–2018. 20719860 10.1210/me.2010-0001 PMC2954635

[15] Mueller S, Kleinau G, Szkudlinski MW, Jaeschke H, Krause G, et al (2009) The superagonistic activity of bovine thyroid-stimulating hormone (TSH) and the human TR1401 TSH analog is determined by specific amino acids in the hinge region of the human TSH receptor. J Biol Chem 284: 16317–16324. 19386596 10.1074/jbc.M109.005710 PMC2713536

[16] Kleinau G, Mueller S, Jaeschke H, Grzesik P, Neumann S, et al (2011) Defining structural and functional dimensions of the extracellular thyrotropin receptor region. J Biol Chem 286: 22622–22631. 21525003 10.1074/jbc.M110.211193 PMC3121406

[17] Fan QR, Hendrickson WA (2005) Structure of human follicle-stimulating hormone in complex with its receptor. Nature 433: 269–277. 15662415 10.1038/nature03206 PMC5514322

[18] Nunez Miguel R, Sanders J, Chirgadze DY, Furmaniak J, Rees Smith B (2009) Thyroid stimulating autoantibody M22 mimics TSH binding to the TSH receptor leucine rich domain: a comparative structural study of protein-protein interactions. J Mol Endocrinol 42: 381–395. 19221175 10.1677/JME-08-0152

[19] Miguel RN, Sanders J, Blundell TL, Smith BR, Furmaniak J (2005) Comparative modeling of the thyrotropin receptor. Thyroid 15: 746–747. 16178067

[20] Sanders J, Chirgadze DY, Sanders P, Baker S, Sullivan A, et al (2007) Crystal structure of the TSH receptor in complex with a thyroid-stimulating autoantibody. Thyroid 17: 395–410. 17542669 10.1089/thy.2007.0034

[21] Sanders J, Oda Y, Roberts SA, Maruyama M, Furmaniak J, et al (1997) Understanding the thyrotropin receptor function-structure relationship. Baillieres Clin Endocrinol Metab 11: 451–479. 9532334 10.1016/s0950-351x(97)80693-3

[22] Oda Y, Sanders J, Roberts S, Maruyama M, Kato R, et al (1998) Binding characteristics of antibodies to the TSH receptor. J Mol Endocrinol 20: 233–244. 9584838 10.1677/jme.0.0200233

[23] Sanders P, Young S, Sanders J, Kabelis K, Baker S, et al (2011) Crystal structure of the TSH receptor (TSHR) bound to a blocking-type TSHR autoantibody. J Mol Endocrinol 46: 81–99. 21247981 10.1530/JME-10-0127

[24] Parker CE, Papac DI, Trojak SK, Tomer KB (1996) Epitope mapping by mass spectrometry: determination of an epitope on HIV-1 IIIB p26 recognized by a monoclonal antibody. J Immunol 157: 198–206. 8683115

[25] Ando T, Latif R, Pritsker A, Moran T, Nagayama Y, et al (2002) A monoclonal thyroid-stimulating antibody. J Clin Invest 110: 1667–1674. 12464672 10.1172/JCI16991 PMC151640

[26] Sanders J, Evans M, Premawardhana LD, Depraetere H, Jeffreys J, et al (2003) Human monoclonal thyroid stimulating autoantibody. Lancet 362: 126–128. 12867115 10.1016/s0140-6736(03)13866-4

[27] Ando T, Latif R, Daniel S, Eguchi K, Davies TF (2004) Dissecting linear and conformational epitopes on the native thyrotropin receptor. Endocrinology 145: 5185–5193. 15297445 10.1210/en.2004-0789

[28] Latif R, Ando T, Davies TF (2007) Lipid rafts are triage centers for multimeric and monomeric thyrotropin receptor regulation. Endocrinology 148: 3164–3175. 17412816 10.1210/en.2006-1580

[29] Hamidi S, Chen CR, McLachlan SM, Rapoport B (2011) Insight into thyroid-stimulating autoantibody interaction with the thyrotropin receptor N-terminus based on mutagenesis and re-evaluation of ambiguity in this region of the receptor crystal structure. Thyroid 21: 1013–1020. 21834684 10.1089/thy.2011.0147 PMC3162645

[30] Morshed SA, Ando T, Latif R, Davies TF (2010) Neutral antibodies to the TSH receptor are present in Graves’ disease and regulate selective signaling cascades. Endocrinology 151: 5537–5549. 20844004 10.1210/en.2010-0424 PMC2954721

[31] Weetman AP, McGregor AM (1994) Autoimmune thyroid disease: further developments in our understanding. Endocr Rev 15: 788–830. 7705281 10.1210/edrv-15-6-788

[32] Benjamin DC, Perdue SS (1996) Site-Directed Mutagenesis in Epitope Mapping. Methods 9: 508–515. 8812706 10.1006/meth.1996.0058

[33] Costagliola S, Bonomi M, Morgenthaler NG, Van Durme J, Panneels V, et al (2004) Delineation of the discontinuous-conformational epitope of a monoclonal antibody displaying full in vitro and in vivo thyrotropin activity. Mol Endocrinol 18: 3020–3034. 15319453 10.1210/me.2004-0231

[34] Cornelis S, Uttenweiler-Joseph S, Panneels V, Vassart G, Costagliola S (2001) Purification and characterization of a soluble bioactive amino-terminal extracellular domain of the human thyrotropin receptor. Biochemistry 40: 9860–9869. 11502179 10.1021/bi0107389

[35] Szkudlinski MW, Fremont V, Ronin C, Weintraub BD (2002) Thyroid-stimulating hormone and thyroid-stimulating hormone receptor structure-function relationships. Physiol Rev 82: 473–502. 11917095 10.1152/physrev.00031.2001

[36] Moyle WR, Lin W, Myers RV, Cao D, Kerrigan JE, et al (2005) Models of glycoprotein hormone receptor interaction. Endocrine 26: 189–205. 16034173 10.1385/ENDO:26:3:189

[37] Costagliola S, Panneels V, Bonomi M, Koch J, Many MC, et al (2002) Tyrosine sulfation is required for agonist recognition by glycoprotein hormone receptors. EMBO J 21: 504–513. 11847099 10.1093/emboj/21.4.504 PMC125869

[38] Chen CR, Salazar LM, McLachlan SM, Rapoport B (2012) Novel information on the epitope of an inverse agonist monoclonal antibody provides insight into the structure of the TSH receptor. PLoS One 7: e31973. 22359649 10.1371/journal.pone.0031973 PMC3281106

[39] Parker CE, Tomer KB (2002) MALDI/MS-based epitope mapping of antigens bound to immobilized antibodies. Mol Biotechnol 20: 49–62. 11876299 10.1385/MB:20:1:049

[40] Raska CS, Parker CE, Dominski Z, Marzluff WF, Glish GL, et al (2002) Direct MALDI-MS/MS of phosphopeptides affinity-bound to immobilized metal ion affinity chromatography beads. Anal Chem 74: 3429–3433. 12139050 10.1021/ac0111199

[41] Raska CS, Parker CE, Sunnarborg SW, Pope RM, Lee DC, et al (2003) Rapid and sensitive identification of epitope-containing peptides by direct matrix-assisted laser desorption/ionization tandem mass spectrometry of peptides affinity-bound to antibody beads. J Am Soc Mass Spectrom 14: 1076–1085. 14530088 10.1016/S1044-0305(03)00405-7

