# Supplementary figures and images for: Antibody Protection Reveals Extended Epitopes on the Human TSH Receptor
Source: PLoS One. 2012 Sep 5;7(9):e44669. doi: 10.1371/journal.pone.0044669 (PMC3434159; doi:10.1371/journal.pone.0044669)

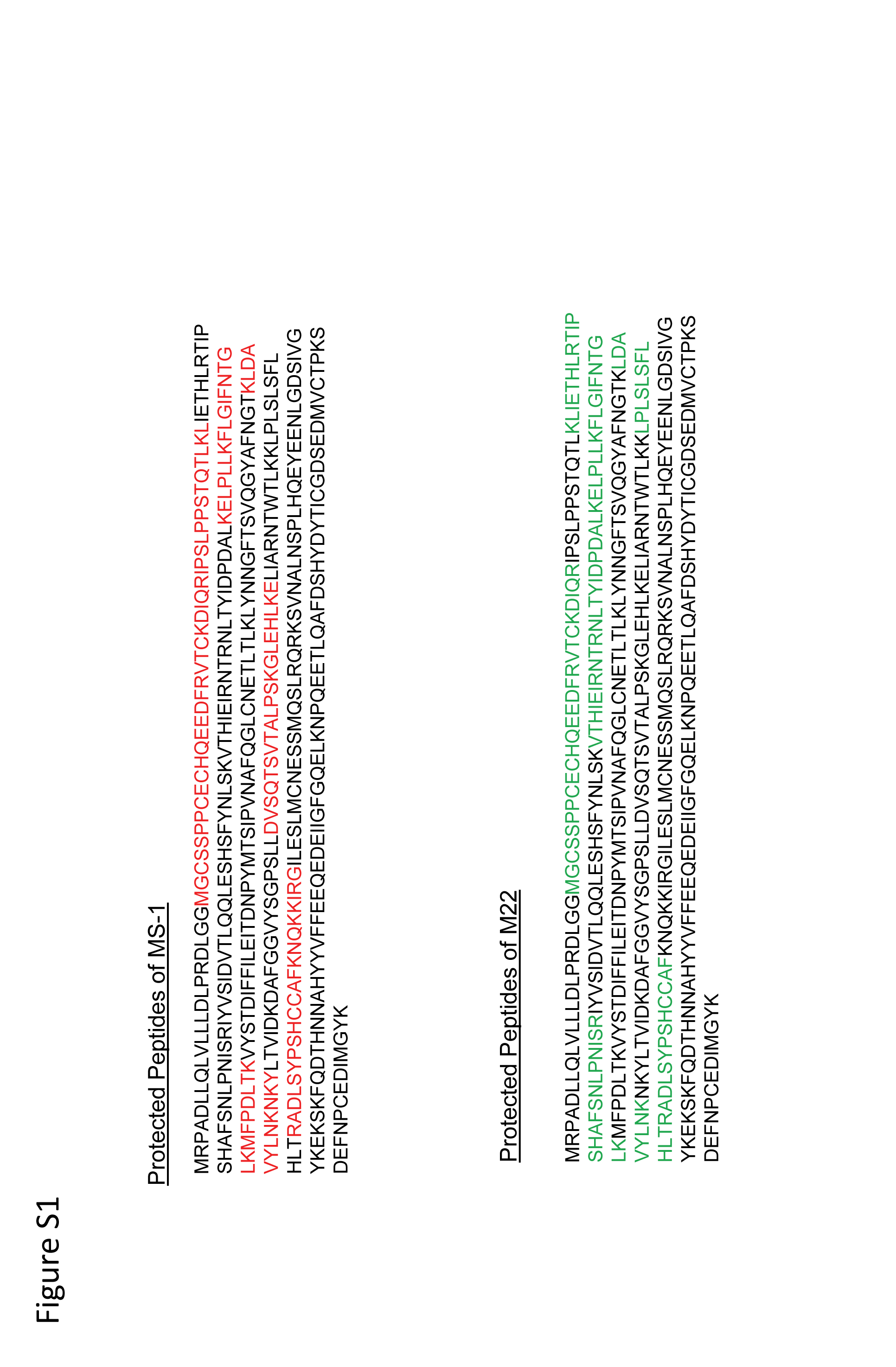

Supplement: Figure S1 — The peptide sequence of the entire TSH receptor ECD is shown in black letters in the top and bottom panels. The MS-1 protected peptides shown in the top panel (marked in red) and M22 protected peptide sequences shown in bottom panel (marked in green). (TIF) [file pone.0044669.s001.tif]
